# Incidence of adjacent segment degeneration in cervical disc arthroplasty versus anterior cervical decompression and fusion meta-analysis of prospective studies

**DOI:** 10.1007/s00402-014-2125-2

**Published:** 2014-11-26

**Authors:** Jiaquan Luo, Ming Gong, Sheng Huang, Ting Yu, Xuenong Zou

**Affiliations:** Department of Spine Surgery/Orthopaedic Research Institute, The First Affiliated Hospital of Sun Yat-sen University, Guangzhou, 510080 People’s Republic of China

**Keywords:** TDR, ACDF, ASD

## Abstract

**Purpose:**

To evaluate the incidence of adjacent segment disease (ASD) requiring surgical intervention between anterior cervical decompression and fusion (ACDF) and total disc replacement (TDR).

**Background:**

The concern for ASD has led to the development of motion-preserving technologies such as TDR. However, whether replacement arthroplasty in the spine achieves its primary patient-centered objective of lowering the frequency of adjacent segment degeneration is not verified yet.

**Methods:**

A comprehensive literature search was performed using PubMed, Cochrane Central Register of Controlled Trials and Embase. These databases were thoroughly searched for prospective randomized studies comparing ACDF and TDR. Eight studies met the inclusion criteria for a meta-analysis and were used to report an overall rate of ASD for both ACDF and TDR.

**Results:**

Pooling data from 8 prospective studies, the overall sample size at baseline was 1,726 patients (889 in the TDR group and 837 in the ACDF group). The ACDF group had significantly more ASDs compared with the TDR group at 24 months postoperatively [odds ratios (OR), 1.31; 95 % confidence interval (CI), 1.04–1.64; *p* = 0.02]. The TDR group had significantly fewer adjacent segment reoperations compared with the ACDF group at 24 months postoperatively (OR, 0.49; 95 % CI, 0.25–0.96; *p* = 0.04).

**Conclusions:**

For patients with one-level cervical degenerative disc disease (CDDD), total disc replacement was found to have significantly fewer ASDs and reoperations compared with the ACDF. Cervical replacement arthroplasty may be superior to ACDF in ASD. Therefore, cervical arthroplasty is a safe and effective surgical procedure for treating CDDD. We suggest adopting TDR on a large scale.

## Introduction

Symptomatic myelopathy and/or radiculopathy are common indications for surgical intervention in the cervical spine. Anterior cervical decompression and fusion (ACDF) has been widely performed and considered as the standard surgical treatment for cervical degenerative disc disease (CDDD). Fusion of the cervical spine has biomechanical consequences. Loss of motion at the operated spinal unit increases the load sustained by the adjacent units [1]. Previous study has demonstrated that anterior cervical fusion is associated with the adjacent segment degeneration (ASD) [2]. However, the cause for ASD remains widely controversial. Some scholars believe that incidence represents a natural progression of cervical disc disease, whereas others suggest altered biomechanics at levels adjacent to a fusion accelerate this process [3, 4].

Cervical disc prostheses are designed to preserve motion patterns and disc height, to avoid the limitations of fusion, and to maintain normal segmental lordosis after surgery. Previous studies have demonstrated that artificial disc arthroplasty offers the theoretical advantage of preservation of motion at the operative level with consequent stress reduction at adjacent levels [5, 6]. However, few clinical studies have specifically aimed to evaluate adjacent segment degeneration after cervical disc arthroplasty.

Whether cervical replacement arthroplasty will achieve its original patient-centered goals with improved outcomes and less adjacent segment degeneration remains an unresolved issue. To further clarify this debate, we perform a meta-analysis of the current available evidence comparing the reported incidence of ASD requiring surgical intervention between ACDF and TDA. This study also aims to emphasize the importance of reporting ASD as an outcome in future prospective studies.

## Materials and methods

### Search strategy

We searched for randomized controlled trials (RCTs) published between January 1960 and June 2014 that compared cervical arthroplasty with ACDF in patients with cervical radiculopathy or myelopathy. The databases included PubMed, Cochrane Central Register of Controlled Trials, and Embase with no language restriction. In addition, we also performed handsearching of information in the Orthopedics China Biological Medicine Database. The following search terms were used: “cervical disc replacement”, “disc replacement”, “cervical artificial disc replacement”, “cervical disc arthroplasty”, and RCT.

### Inclusion criteria

The inclusion criteria were: (1) randomized, controlled study of degenerative disc disease of the cervical spine involving single segment or double segments using CDA with anterior cervical discectomy and fusion (ACDF) as controls; (2) a minimum of 2-year follow-up using imaging and clinical analyses; (3) definite diagnostic evidences for “adjacent segment degeneration” and “adjacent segment disease”.

### Exclusion criteria

Exclusion criteria were: (1) case reports; (2) reviews; (3) patients with cervical spine disease involving more than three segments.

### Study selection

Two of the authors (J.-Q.L. and S.H.) independently screened the article titles and abstracts based on the eligibility criteria. Intensive reading of the full text was performed when the studies met the inclusion criteria. Disagreements were resolved by discussion to reach a consensus.

### Data extraction

Relevant data were extracted independently by two authors (J.-Q.L. and M.G.). The data included the general characteristics of each study and the outcomes measured. General characteristics included study design, first author, year of publication, sample size, interventions and various types of artificial total disc replacements (TDRs). The outcomes measured included: the rate of postoperative development of adjacent segment degenerative or diseases and the rate of adjacent segment surgery. Discrepancies were resolved through discussion.

### Quality assessment

According to the Cochrane Handbook for Systematic Reviews of Interventions, version 5.0, the quality of the studies was independently evaluated by two authors (J.-Q.L. and T.-Y.). The following domains were assessed: randomization, blinding (of patients, surgeons, and assessors), allocation concealment, and follow-up coverage. Each domain of quality assessment was classified as adequate (A), unclear (B), or inadequate (C).

### Data analysis

We performed all meta-analyses with the Review Manager software (RevMan Version 5.1; The Nordic Cochrane Center, The Cochrane Collaboration, Copenhagen, Denmark). Only dichotomous outcomes were mentioned in our study, so the OR or risk ratios and 95 % confidence intervals were calculated for outcomes. A probability of *p* < 0.05 was considered to be statistically significant. Assessment for statistical heterogeneity was calculated using the Chi-square and *I*-square tests. *I* ^2^ ranges from 0 to 100 %, with 0 % indicating the absence of any heterogeneity. Although absolute numbers for *I* ^2^ are not available, values <50 % are considered low heterogeneity. When *I* ^2^ is <50 %, low heterogeneity is assumed, and the effect is thought to be due to change. Conversely, when *I* ^2 > ^50 %, heterogeneity is thought to exist and the effect is random.

## Results

**Fig. 1:**
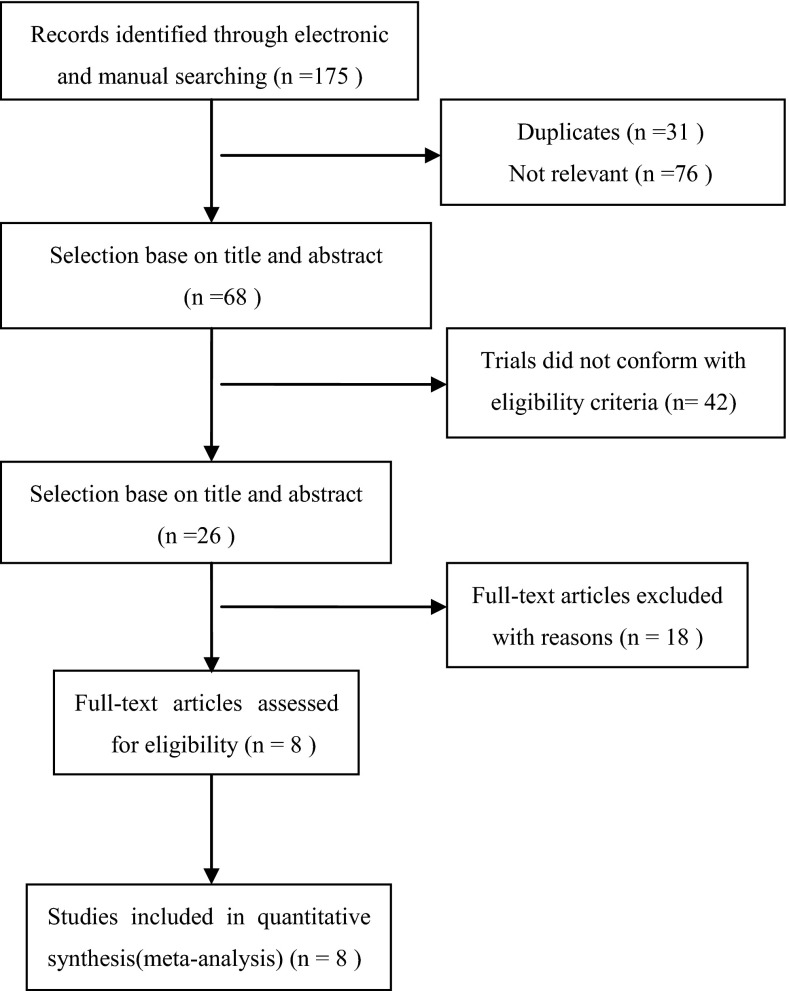
The flow chart shows the article selection process we performed

The process of identifying relevant studies is summarized in Fig. 1. From the selected databases, 175 references were obtained. By screening the titles and abstracts, 107 references were excluded due to the duplicates and irrelevance to this topic. The remaining 68 reports underwent a detailed and comprehensive evaluation. Finally, 8 RCTs were included in this meta-analysis [7–14]. The main characteristics of included studies are summarized in Table 1.

**Table 1:** Characteristics of studies included in the meta-analysis of cervical arthroplasty compared to anterior cervical discectomy and fusion for treating one-level cervical disc disease

Studies	Design	Country	Sample size		Mean age (years)		Gender (M/F)		Follow-up (months)
			TDR	ACDF	TDR	ACDF	TDR	ACDF	
Porchet [7]	RCT 4 centers	Switzerland	27	28	44 ± 8.9	43 ± 6.9	17/10	12/16	24
Murrey [8]	RCT 13 centers	USA	103	106	42.1 ± 8.4	43.5 ± 7.1	46/57	49/57	24
Nabhan [9]	RCT 1 centers	Germany	20	21	44		23/18		36
Garrido [10]	RCT 1 centers	USA	21	26	40	43.3	13/8	26/14	48
Burkus [11]	RCT 32 centers	USA	276	265	43.3	43.9	128/148	122/143	60
Sasso [12]	RCT 31 centers	USA	242	221	44.4 (25–78)	44.7 (27–68)	110/132	113/108	48
Coric [13]	RCT 21 centers	USA	136	133	43.7 ± 7.76	43.9 ± 7.39	51/85	59/74	24
Jawahar [14]	RCT 1 centers	USA	59	34	–	–	21/38	16/18	24

Mean age was described as mean ± SD or mean (range)

*ACDF* anterior cervical discectomy and fusion, *RCT* randomized control trial, *SD* standard deviation, *TDR* total disc replacement, *M* male, *F* female

### Quality assessment

The results of the quality assessment are presented in Table 2. Of the eight studies, eight stated the exact randomization methods used [7–14]. Two studies blinded both the patients and the assessors [12, 14]. None of the studies documented concealment of randomization. Demographic data at baseline were similar in the two treatment groups. Descriptions of patient drop-outs and withdrawals appeared in all eight reports. Hence, the methodological quality of the eight studies included was level B.

**Table 2:** Methodological quality of studies included in the meta-analysis of cervical arthroplasty compared to ACDF for treating one-level CDDD

Years	Baseline			Randomization	Allocation concealment	Blinding	Quality level
	Size	Age	Sex				
Porchet [7]	Comparable	Comparable	Comparable	Adequate	Unclear	Unclear	B
Murrey [8]	Comparable	Comparable	Comparable	Adequate	Unclear	Unclear	B
Nabhan [9]	Comparable	Comparable	Comparable	Adequate	Unclear	Unclear	B
Garrido [10]	Comparable	Comparable	Comparable	Adequate	Unclear	Unclear	B
Burkus [11]	Comparable	Comparable	Comparable	Adequate	Unclear	Unclear	B
Sasso [12]	Comparable	Comparable	Comparable	Adequate	Unclear	Double	B
Coric [13]	Comparable	Comparable	Comparable	Inadequate	Unclear	Single	B
Jawahar [14]	Comparable	Comparable	Comparable	Adequate	Unclear	Double	B

Comparable: the variables were comparable among all studies

Each domain of quality assessment was classified as: adequate (A), unclear (B), or inadequate (C)

### Surgical parameters

#### Adjacent segment disease

**Fig. 2:**
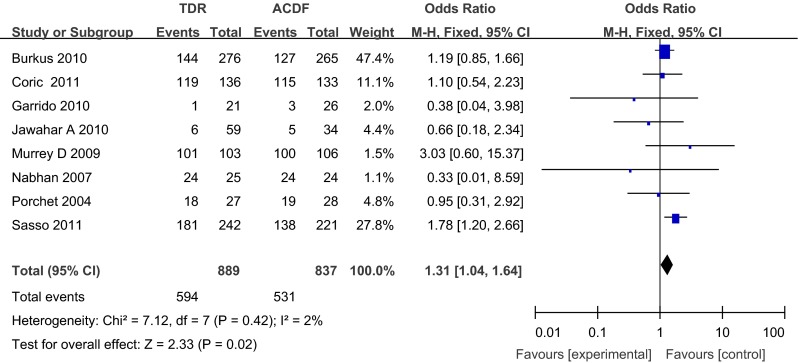
Forest plot of adjacent segment disease for the TDR and ACDF groups at 24 months postoperatively. *ACDF* anterior cervical discectomy and fusion, *TDR* total disc replacement, *CI* confidence interval, *M–H* Mantel–Haenszel, *SD* standard deviation

Adjacent segment disease was provided in 8 studies, and all these studies with a total of 1,726 patients (889 in the TDR group and 837 in the ACDF group) were analyzed. The ACDF group had significantly more adjacent segment diseases compared with the TDR group at 24 months postoperatively (OR, 1.31; 95 % CI, 1.04–1.64; *p* = 0.02) (Fig. 2).

#### Adjacent segment reoperations

**Fig. 3:**
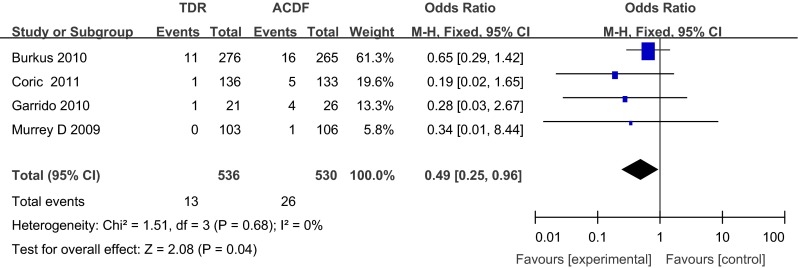
Forest plot of adjacent segment reoperations for the TDR and ACDF groups at 24 months postoperatively. *ACDF* anterior cervical discectomy and fusion, *TDR* total disc replacement, *CI* confidence interval, *M–H* Mantel–Haenszel, *SD* standard deviation

Adjacent segment reoperations were provided in 4 studies with a total of 1,066 patients (536 in the TDR group and 530 in the ACDF group) analyzed. The TDR group had significantly fewer adjacent segment reoperations compared with the ACDF group at 24 months postoperatively (OR, 0.49; 95 % CI, 0.25–0.96; *p* = 0.04) (Fig. 3).

## Discussion

In this meta-analysis, we identified 8 randomized clinical trials with up to minimum 24 months of follow-up assessing the effects of TDR for patients with one-level CDDD refractory to nonoperative treatment. We found that the TDR group had significantly fewer adjacent segment diseases and reoperations compared with the ACDF.

A few meta-analyses have been published recently comparing ACDF versus TDR. Results of our meta-analyses showed that TDR group had significantly fewer adjacent segment reoperations compared with the ACDF group at 24 months postoperatively. Gao et al. [15] reported that arthroplasty was associated with fewer secondary surgical procedures. However, the indications for these secondary procedures were unclear. From the 2-year follow-up data, Phillips et al. [16] reported an equivalent rate of secondary procedures for ASD versus TDR (5.4 versus 5.2 %). However, the study does not specifically report the number of reoperations for ASD alone.

With regard to ASD, our meta-analysis showed that the ACDF group had significantly more ASD compared with the TDR group at 24 months postoperatively.

A recent meta-analysis by Jiang et al. [17] also found a lower rate of ASD for TDR versus ACDF. However, this analysis included radiographical assessments of ASD that do not correlate with reoperation rate. In addition, the analysis was heavily influenced by the 2-year follow-up data from Mummaneni et al. [18]. The 5-year data from this same author, however, showed equivalent ASD rates for ACDF versus TDR [19]. Verma et al. concluded there is no difference in the rate of ASD for ACDF versus TDA. They report an overall lower rate of follow-up for patients with ACDF than for those with TDR [20]. Similarly, Yang et al. reported no difference in the incidence of ASD (radiographical features and reoperation rate) comparing ACDF versus TDR in a meta-analysis. Although the conclusion of the authors was similar to that of this study, there were methodological differences worth nothing [21]. In addition, Yang et al. [21] included only 140 patients in the meta-analysis, whereas this study included more than 1,500 patients at baseline and 1,100 patients at the final follow-up. Lastly, the meta-analysis by Yang et al. [21] was update to 2011, but their selection of studies was notably different than that of this study.

There are many and complicated reasons for developing ASD after ACDF and arthroplasty, such as the increased adjacent vertebral sagittal activity [22], the fusion segment number [23], the segment locations [23], segmental kyphosis operation [24], and the influence of each factor on the other. Increased stress of fused adjacent segments is the reason of causing ASD [25]. A biomechanical and kinematic study suggested that preservation of motion at the operated level might help to lessen the incidence of adjacent-level disc degeneration [26]. TDR is developed to restore physiologic biomechanics and to reduce the adjacent-level forces, thereby reducing the potential for accelerated adjacent-level disc degeneration [27]. However, whether cervical replacement arthroplasty will achieve its original patient-centered goals with improved outcomes and less adjacent segment degeneration remains unclear.

In our meta-analysis, eight published RCTs on cervical TDR versus fusion were analyzed. Seven studies had good methodological qualities (Jadad scores ≥ 4); one study only gained three scores which implied a higher risk of bias. The most prevalent methodological shortcoming appeared to be insufficiency regarding the outcome assessor blinding to intervention. The low number of included studies limited our assessment of potential publication bias by the funnel plot and unpublished researches with negative results cannot be identified. Therefore, publication bias may exist, which could result in the overestimation of the effectiveness of interventions.

We believe that our result of meta-analysis is affected by several reasons. First, the number of articles may be insufficient and we included only eight studies in the evaluation, what might have led to an insufficient significant effectiveness. Second, the low number of included studies limited our assessment of a potential publication bias which cannot be excluded due to unpublished negative research results. Therefore, publication bias may exist, which might have resulted in the overestimation of the intervention effectiveness. Third, the properties of the different prostheses, the various indications for surgery, and the surgical technologies used at different treatment centers. Due to these limitations, the combined results of this meta-analysis should be cautiously accepted, and high-quality RCTs with long-term follow-up and large sample size are needed.

In summary, our meta-analysis indicated, for the treatment of CDDD, that cervical disc arthroplasty had significantly fewer adjacent segment diseases and reoperations compared with the ACDF. TDR may be superior to ACDF in ASD.

## Conclusion

For patients with one-level CDDD, TDR was found to have significant fewer adjacent segment diseases and reoperations compared with the ACDF. TDR may be superior to ACDF in ASD. Therefore, TDR is a safe and effective surgical procedure for treating CDDD. We suggest adopting TDR on a large scale.
